# DLL3 expression in neuroendocrine carcinomas: a comparative analysis across tumor origins

**DOI:** 10.3389/fendo.2026.1864568

**Published:** 2026-08-12

**Authors:** Chiara Calabrese, Sofia Dellavalle, Chiara Spadazzi, Beate Sable, Dorothy French, Kumiko Isse, Khushboo Sharma, Vincenzo Laino, Roberto Fasano, Maria Maddalena Tumedei, Federica Pieri, Nicoletta Ranallo, Flavia Foca, Alessandro De Vita, Giacomo Miserocchi, Claudia Cocchi, Silvia Vanni, Sofia Gabellone, Oneda Cani, Giulia Sbanchi, Alberto Bongiovanni, Chiara Liverani

**Affiliations:** 1 Preclinic and Osteoncology Unit, Biosciences Laboratory, IRCCS Istituto Romagnolo per lo Studio dei Tumori (IRST) “Dino Amadori”, Meldola, Italy; 2 Amgen, Inc., Thousand Oaks, CA, United States; 3 Advanced Cellular Therapies and Rare Tumors Unit, IRCCS Istituto Romagnolo per lo Studio dei Tumori (IRST) “Dino Amadori”, Meldola, Italy; 4 Pathology Unit, “Morgagni-Pierantoni” Hospital, Forlì, Italy; 5 Clinical and Experimental Oncology, Immunotherapy, Rare Cancers and Biological Resource Center, IRCCS Istituto Romagnolo per lo Studio dei Tumori (IRST) “Dino Amadori”, Meldola, Italy; 6 Biostatistics and Clinical Trial Unit, IRCCS Istituto Romagnolo per lo Studio dei Tumori (IRST) “Dino Amadori”, Meldola FC, Italy; 7 Departmental Faculty of Medicine, UniCamillus-Saint Camillus International University of Health and Medical Sciences, Rome, Italy

**Keywords:** biomarkers, DLL3, immunohistochemical, neuroendocrine carcinomas (NECs), patients

## Abstract

**Background:**

Neuroendocrine carcinomas (NECs) are rare and highly aggressive tumors, characterized by a dismal prognosis. Their underlying biology remains insufficiently understood, highlighting an urgent need for the identification of disease markers to enhance diagnostic and prognostic assessments, as well as to identify potential druggable targets. DLL3, an inhibitory regulator of the Notch signalling pathway, has emerged as a promising candidate in this context.

**Methods:**

We investigated DLL3 prevalence and pattern of expression in immunohistochemistry in NECs from multiple anatomical sites, with a focus on lung and gastroenteropancreatic (GEP) neoplasms.

**Results:**

DLL3 was found to be highly expressed in 68.8% of NECs overall, with the highest prevalence observed in GEP-NECs (85.7%). Immunohistochemical analysis revealed predominantly cytoplasmic localization, with a dot-like staining pattern particularly evident in gastrointestinal cases, suggestive of potential localization in neurosecretory granules. DLL3 levels did not significantly correlate with tumor proliferation index nor with overall survival. A correlation with chromogranin A expression was found.

**Conclusions:**

Given its high prevalence in extrapulmonary NECs, particularly those of GEP origin, it may have potential as a disease marker and therapeutic target in a clinical context where effective options are currently limited.

## 1 Introduction

Neuroendocrine neoplasms (NENs) are rare cancers characterized by heterogeneous biological and clinical features. They are divided into well-differentiated neuroendocrine tumors (NETs) and poorly differentiated neuroendocrine carcinomas (NECs) (1). NECs display high proliferative index, significant necrosis and nuclear atypia, and can be classified as small cell, large cell, and mixed histologies. They can occur in different anatomical sites with the lung being the most prevalent (2). Extrapulmonary NECs (EP-NECs) frequently arise in the gastroenteropancreatic (GEP) tract (~37%), followed by the genitourinary (~17%) and gynecological tracts (~10%) (3, 4). Most lung NECs exhibit small cell morphology, whereas GEP-NECs are predominantly large cell, except in specific sites such as the esophagus, gallbladder, and anal canal (5, 6). Diagnosis relies on immunohistochemical markers including synaptophysin, chromogranin-A, insulinoma-associated protein 1 (INSM1), and somatostatin receptors (SSTRs), while transcription factors such as TTF1, CDX2, and ISL-1 help determine the site of origin, particularly in metastatic cases (7). Chromogranin A is widely regarded as one of the most reliable biomarkers for NETs. However, in contrast to well-differentiated NETs, NECs often show variable chromogranin A expression in both extent and intensity. GEP-NETs are further divided according to their proliferative index into Grade 1 (G1, Ki-67 <3%), Grade 2 (G2, Ki-67 3–20%), and Grade 3 (G3, Ki-67 >20%) (8). Although G3-NETs have better clinical outcomes and distinct chemotherapy responses than NECs, no validated objective criteria are currently available for the distinction of these two entities. Diagnosis largely depends on the assessment of differentiation, despite efforts to identify potential biomarkers. A mutation-based classification of TP53, APC, KRAS, and BRAF, has been recently proposed, providing 83% positive predictive value for differentiating large cell NECs from G3 NETs (9). Additionally, an immunohistochemical study by Kanaan et al. found p53 and Rb expressions to be strongly associated with NECs rather than G3-NETs (10). EZH2 has also been found to be predominantly overexpressed in GEP and pulmonary NECs (11). However, none of these findings have yet led to significant changes in clinical diagnostic practice.

NEC patients typically present with multiple metastases and show poor survival (1, 2, 8). Treatment options are limited with few drugs entering clinical use despite the number of trials (12, 13). Platinum/etoposide-based chemotherapy remains the first-line treatment, offering response rates of 41–67% but short duration, with median progression-free survival (mPFS) of 9 months and median overall survival (mOS) of 15–19 months (14). Identifying new drug targets is therefore critically needed. The Notch pathway has been identified as a crucial factor in both primary and secondary neuroendocrine tumor development (15, 16). Additionally, DLL3 (Delta-like ligand 3), an inhibitory ligand of Notch receptors, is highly expressed in several neuroendocrine tumors (17–20). DLL3 relevance in NENs was first noted in lung tumors, where its expression serves as a distinctive molecular marker for small cell lung cancer (SCLCs) and a subtype of large cell NECs (LCNECs) (21). In SCLC, DLL3 expression correlates with disease progression and may help in grade distinction (22). In neuroendocrine prostate cancer, it links to aggressive features, RB1 loss, and it has been detected in circulating tumor cells (19). As a result, there has been increasing interest in developing targeted treatments for DLL3-expressing tumors. This has led to the clinical evaluation of the first DLL3-targeted chimeric antigen receptor T (CAR-T) cell therapy (AMG 119) in SCLC (23) and the approval of tarlatamab, a bispecific T-cell engager targeting DLL3 and CD3, in formerly treated SCLCs (24–26). Tarlatamab has also been investigated in SCLC as a first-line maintenance treatment in combination with immunotherapy, with outcome results supporting an ongoing Phase 3 trial (NCT06211036) (27). Recently, we reported the first evidence of DLL3 protein expression in GEP-NEC cells and demonstrated that NECs and NETs can be distinguished based on DLL3 protein expression (28). Here, we analyzed the expression patterns of DLL3 in a retrospective case series of patients with NECs originating from various anatomical sites with the aim to define the prevalence of this biomarker across different subtypes of NECs.

## 2 Materials and methods

### 2.1 Study design

This is a retrospective, single-center biological study conducted on histological or bioptic samples obtained from patients diagnosed with NEC. The protocol was reviewed and approved by the local ethical committee. Patients eligible for the study were adults (at least 18 years of age) of both sex, with a histologically confirmed diagnosis of NEC of all sites of origin, and had to sign a written informed consent. The histologies were revised by an experienced pathologist. For patients who could not be re-contacted, the Principal Investigator subscribed to a Declaration of Anonymization (provisions No. 146 of June 5, 2019). For all patients enrolled, we collected biological and clinical data including Ki67, sites of disease and mOS.

### 2.2 Immunohistochemical staining

Unstained 5-μm-thick microtome sections of formalin-fixed paraffin-embedded (FFPE) tumor samples from NEC patients were collected using a rotating microtome (Leica Biosystems, Wetzlar, Germany) and were mounted on positive-charged microslides (Thermo Fisher Scientific,Waltman, MA, USA). Staining was performed on the VENTANA BenchMark Ultra (Ventana Medical Systems Inc, Tucson, AZ, USA). The DLL3 (SP347) Ventana Assay, prediluted by the supplier, was used. The reactions were carried out for 1 h at room temperature, and sections were counterstained with Hematoxylin II (Ventana Medical System). DLL3 expression was evaluated by an expert pathologist. Stained sections were digitized using the Aperio scanning system (Leica Biosystems). For Ki-67 and chromogranin evaluation, the following antibodies were used: Ki-67 Antigen (Dako Omnis) Clone MIB-1 1:100; Anti-Chromogranin A Primary Antibody prediluted by the supplier (Ventana Medical Systems Inc.); Anti-Synaptophysin Primary Antibody prediluted by the supplier (Ventana Medical Systems Inc.) clone SP11. Staining was performed using the VENTANA BenchMark Ultra automated system, following the routine clinical diagnostic protocol.

### 2.3 Evaluation of DLL3 expression

DLL3 expression and scoring was performed according to the manufacturer’s validated guidelines, by a trained pathologist, with an experience in the diagnostic assessment of NENs. To ensure accuracy, at least 100 viable tumor cells with a well-preserved morphology were considered. Necrotic tissue areas and artifacts were excluded. DLL3 expression was evaluated considering both membrane and cytoplasmic staining patterns. Cases with no staining or weak (1+) staining led to a negative classification according to established criteria (29), while cases with moderate (2+) or strong (3+) staining were considered positive. Cases were classified based on the highest detected staining intensity; thus, cases with both moderate and weak staining were categorized as moderate. A positivity percentage was assigned, calculated as the ratio of the number of cells exhibiting moderate or strong staining to the total number of cells. The ratio was calculated and multiplied by 100 to obtain the percentage. Staining localization (membrane, cytoplasm or both) was systematically recorded during the evaluation.

### 2.4 Statistical analysis

Categorical variables were summarized using frequencies and percentages, while continuous variables were reported as median and minimum-maximum values. Overall survival (OS) was calculated from the date of diagnosis to the date of death. Survival probabilities were estimated using the Kaplan-Meier method, and survival curves were generated to visualize differences between groups. To evaluate the impact of DLL3 and the main clinicopathological variables including metastatic disease at diagnosis and primary tumor site as potential prognostic factors on survival, the log-rank test was performed. To account for potential confounding factors, a multivariable Cox proportional hazards regression model was also constructed. To evaluate the association between DLL3 expression and the markers Ki-67 and chromogranin A, the Fisher exact test was performed. A p-value < 0.05 was considered statistically significant. All analyses were performed using Stata/SE version 15.1 for Windows (StataCorpLP, College Station, TX, USA).

## 3 Results

### 3.1 Clinical and biological characteristics of enrolled patients

The main clinical and biological characteristics of the 48 patients analyzed in this study are shown in **Table 1**. Median age at the time of diagnosis was 71 years (range 42–85 years). Thirty patients (62.5%) were males, and eighteen (37.5%) were females. The origin site of primary tumor was lung in 24 patients (50%), the GEP trait (stomach, colon, pancreas, bile ducts and gallbladder) in 14 patients (29.2%), head and neck district (paranasal sinuses, parotid, retropharynx, larynx) in 4 patients (8.2%), uterus in 2 patients (4.2%), bladder in 2 patients (4.2%), unknown in 2 patients (4.2%). Ki-67 was higher than 55 in 26 patients (76.5%), lower than or equal to 55 in 8 patients (25.0%) and not available in 15 patients.

**Table 1:** Clinical and biological characteristics of patients.

Patient characteristics	*n* (%)
Total cases	48
Median age at diagnosis, years (min-max)	71 (42-85)
Gender	
Male	30 (62.5)
Female	18 (37.5)
Site of disease	
Lung	24 (50.0)
GEP trait	14 (29.2)
Head and neck district	4 (8.2)
Uterus	2 (4.2)
Bladder	2 (4.2)
Unknown	2 (4.2)
Ki-67	
≤ 55	8 (16.6)
> 55	26 (54.1)
NA	14(29.2)
Histological features	
Large cell	7 (14.6)
Small cell	41 (85.4)

NA, not available.

Seven patients (14.6%) received a histological diagnosis of large cell NEC, and 41 patients (85.4%) of small cell NEC. Histological diagnosis of all case series and their relative Ki-67 values are reported in **Supplementary Table 1**. Ki-67 was unavailable in 14 cases, primarily among lung NECs, because its assessment was not consistently included in the routine diagnostic work-up during the study period.

### 3.2 Prevalence of DLL3 expression

DLL3 was found to be expressed in 33 out of 48 cases (68.8%), while it was negative in 12 cases (25.0%). In the remaining 3 cases (6.3%), DLL3 evaluation was not considered acceptable due to an insufficient number of cells or to the presence of positivity in the tumor parenchyma, according to guidelines. Taking into consideration the sites of tumor origin, the highest prevalence of DLL3 was found in GEP-NECs. In this subgroup, positive expression was found in 12 out of 14 analyzed cases (85.7%), and only one case was found to be negative (7.1%). In the remaining case, DLL3 evaluation was not considered acceptable due to an insufficient number of cells. In the lung subgroup, positive expression was found in 14 out of 24 cases (58.3%), while 8 cases (33.3%) were found to be negative. In the remaining 2 cases (8.3%), DLL3 evaluation was not considered acceptable due to the presence of positivity in the tumor parenchyma in one case, and to an insufficient number of cells in the other case. In the four NECs originating in the head and neck district, DLL3 was found to be positive in 3 cases (75.0%) and negative in the other case (25.0%). In the two bladder NECs, DLL3 was found to be positive in 1 case (50.0%) and negative in the other case (50.0%). In the two NECs originating in the uterus, DLL3 was found to be positive (100.0%). In the 2 NECs of unknown origin, DLL3 was found to be positive in 1 case (50.0%) and negative in the other case (50.0%). Results are reported in **Table 2**.

**Table 2:** DLL3 expression according to sites of origin.

Site of origin	Positive (%)	Negative (%)	NA	Total
Overall	33 (68.8)	12 (25.0)	3 (6.3)	48
Lung	14 (58.3)	8 (33.3)	2 (8.3)	24
GEP trait	12 (85.7)	1 (7.1)	1 (7.1)	14
Head and neck district	3 (75.0)	1 (25.0)	-	4
Bladder	1 (50.0)	1 (50.0)	-	2
Uterus	2 (100.0)	0 (0.0)	-	2
Unknown primary site	1 (50.0)	1 (50.0)	-	2

### 3.3 Percentage, localization and intensity of DLL3 expression

**Figure 1:**
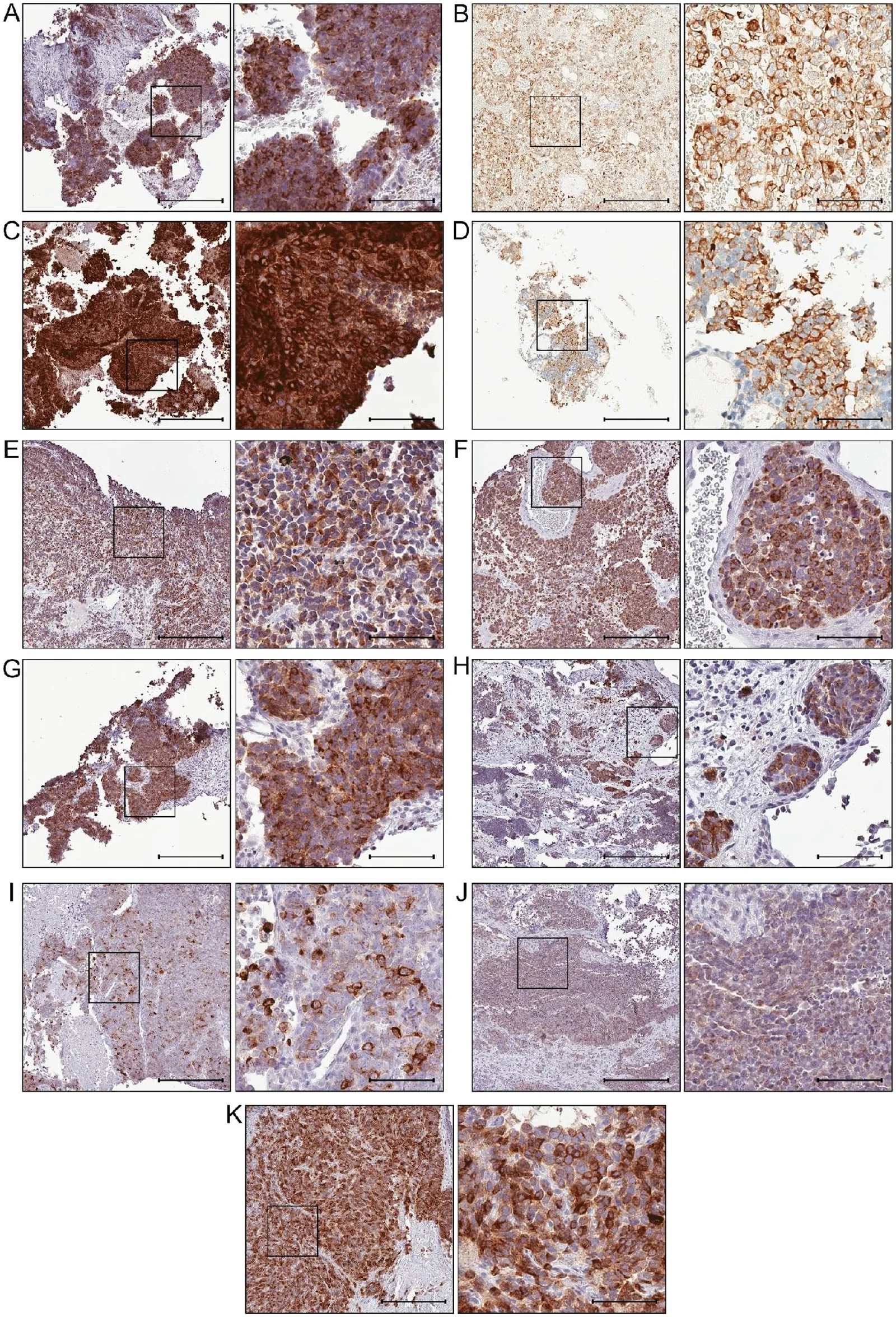
Representative immunohistochemical images of DLL3 expression patterns in NEC cases of various origin sites. **(A)** Pancreas, 80% positivity, **(B)** Stomach, 90% positivity, **(C)** Pancreas, 90% positivity, **(D)** Pancreas, 45% positivity, **(E)** Lung, 80% positivity, **(F)** Lung, 80% positivity, **(G)** Lung, 80% positivity, **(H)** Lung, 30% positivity, **(I)** Retropharynx, 35% positivity, **(J)** Uterus, 20% positivity and **(K)** Unknown origin, 70% positivity. Noticeable differences in expression patterns and staining localization are observed both among different tissue sites and between cases originating from the same site. Images were taken at 10× magnification, with insets magnified to 40×. The scale bar represents 300 µm for 10× images and 60 µm for 40× images.

The overall percentage of positive cells ranged from 3 to 90, showing either a diffuse or clonal pattern. DLL3 was found to be localized in the cytoplasm in 20 cases (60.6%), in the membrane in 5 cases (15.2%), and in both cellular compartments in 8 cases (24.2%). In some of these cases, a dot-like pattern of staining was observed. The different patterns are highlighted in **Supplementary Figure 2**. Intensity of expression was moderate in 27 cases (81.8%) and strong in 6 (18.2%). Results are reported in **Table 3** and **Supplementary Table 2**. **Figure 1** shows representative images of DLL3 expression patterns in different tissue locations. Regarding the GEP trait cases, examples with moderate to strong staining (as in C) are shown, exhibiting predominantly membranous and cytoplasmic distribution. Pulmonary cases also demonstrate heterogeneity in DLL3 localization, with staining present in both cytoplasm and membrane. Additionally, panel G highlights a dot-like pattern, which is also observed in the head and neck case shown in panel I. Panel J depicts a case from the uterine site, characterized by a moderate cytoplasmic staining. A case from an unknown origin is also included, as it is particularly useful for further illustrating the membranous staining pattern.

**Table 3:** DLL3 expression in NEC patient cases.

ID case	Site of origin	DLL3 expression (1 pos, 0 neg)	Percentage of positive cells	Localization	Intensity
144	Lung	NA			
15881	Lung	1	30	M; C	Moderate
2003	Lung	0			
3488	Lung	0			
4775	Lung	1	80	C	Moderate
6213	Lung	0			
6737	Lung	0			
12074	Lung	0			
14220	Lung	0			
8791	Lung	1	80	M; C	Moderate
13528	Lung	1	70-80	C	Moderate
6503	Lung	NA			
2473	Lung	1	60	C	Moderate
12793	Lung	1	30	C	Moderate
11572	Lung	0			
14491	Lung	1	80	C	Moderate
15002	Lung	1	80	C; DL	Moderate
15644	Lung	1	80	C	Moderate
3389	Lung	1	70	C; DL	Moderate
129	Lung	1	80	C	Moderate
6375	Lung	1	80	C	Moderate
1437	Lung	1	60	C	Moderate
5735	Lung	0			
10437	Lung	1	20	C	Moderate
15347	Stomach	1	90	M; DL	Moderate
10907	Stomach	1	35	C; DL	Moderate
11307	Stomach	1	1-10	C; DL	Moderate
2541	Colon	1	3	M; DL	Moderate
11265	Colon	0			
8664	Colon	1	60	C	Moderate
8368	Pancreas	1	60	M; C	Moderate
11211	Pancreas	1	25	M; C	Moderate
7075	Pancreas	1	40/50	M; C	Moderate
198	Pancreas	1	2-10	M; C	Moderate
6902	Pancreas	1	90	M	Strong
4146	Pancreas	1	80	C	Strong
4214	Bile ducts	NA			
12599	Gallbladder	1	20	C	Moderate
8648	Paranasal sinuses	0			
10423	Parotid	1	60	M	Strong
4500	Retropharynx	1	35	C; DL	Strong
8354	Larynx	1	90	M	Strong
5130	Bladder	1	80	C; DL	Moderate
11955	Bladder	0			
9451	Uterus	1	40-50	M; C	Moderate
13693	Uterus	1	70	C	Moderate
9017	Unknown	0			
17191	Unknown	1	70	M; C	Strong

M, membrane; C, cytoplasm; DL, dot like; NA, not available.

### 3.4 Clinical significance of DLL3 expression

To assess the association between mOS and the main clinicopathological variables, including the presence of metastatic disease at diagnosis, site of disease and DLL3 expression we first performed a log-rank test (**Table 4**). At the time of data analysis, all patients were deceased except for one individual. Among these variables, only the presence of distant metastases at diagnosis was significantly associated with worse mOS (9.8 months, 95% CI: 0.9-13.6, *P* = 0.019). The Kaplan–Meier survival analysis showed significantly shorter mOS in patients with metastatic disease (**Supplementary Figure 1**). Neither primary tumor site nor DLL3 expression was significantly associated with mOS. Regarding DLL3 expression, the cases were categorized into three groups based on DLL3 expression: negative, low-positive, and high-positive. Because no validated biological or clinically established cutoff for DLL3 expression is currently available, the median value of expression calculated on the percentages of positive cells was used to dichotomize DLL3 into low-positive (DLL3 lower than the median) and high-positive cases (higher than the median), while the negative group consisted of patients with no expression of DLL3. A median value of 0.6 for DLL3 was observed among positive cases, with 0.03 as minimum and 0.90 as maximum one. The mOS in the overall patient group was 6.6 months (95% CI: 3.6-8.9) (**Table 4**). The mOS for patients with DLL3-negative tumors was 7.3 months (95% CI: 2.1-10.4), compared to 3.6 months (95% CI: 0.9–8.3) in the DLL3 low-positive group, and 8.9 months (95% CI: 0.5–13.4) in the DLL3 high-positive group. These results do not show a clear, linear relationship between DLL3 expression and mOS (P = 0.067). Notably, the lowest mOS was observed in the low-positive group, whereas both negative and high-positive groups showed longer survival. The Kaplan-Meier survival curve indicated that patients with DLL3-negative expression initially exhibit a trend toward improved overall survival compared to other subgroups. However, this survival advantage diminishes over time, with the survival probabilities of the DLL3-negative group gradually converging with those of the DLL3-positive cohorts in the later follow-up period (**Supplementary Figure 1**). These findings may reflect limitations in sample size and the presence of metastasis at diagnosis as a confounding variable In the multivariable model, metastatic disease remained the only independent predictor of worse mOS (HR 3.32, 95% CI 1.37–8.04, P = 0.008) (**Table 5**). Primary tumor site was not significantly associated with survival. Compared with DLL3-negative tumors, patients with high DLL3 expression showed a trend toward improved mOS (HR 0.42, 95% CI 0.17–1.05, P = 0.065), whereas low DLL3 expression was not significantly associated with outcome (HR 2.16, 95% CI 0.84–5.57, P = 0.110). Given that anti-DLL3–targeted therapies are expected to require detectable membrane antigen expression for activity (34, 40), we performed an additional analysis restricted to cases with membranous DLL3 staining. This analysis did not reveal a statistically significant association between membranous DLL3 expression and mOS (**Supplementary Table 3**). Furthermore, we analyzed the relation between DLL3 and Ki-67 (dichotomized into positive or negative) (**Table 6**). Among the 8 patients with Ki-67 value ≤ 55%, 2 were negative for DLL3 (25.0%) and 6 were positive (75.0%), while among the 26 cases with Ki-67 value > 55%, 4 were negative for DLL3 (16.0%) and 21 were positive (84.0%). For 1 patient with Ki-67 > 55%, evaluation of DLL3 resulted not acceptable. No significant association between DLL3 and Ki-67 values was observed (P = 0.456), suggesting that DLL3 expression is independent of the tumor proliferative activity. Additionally, no difference in overall survival was observed among patients with different values for Ki-67 (<= 55% or > 55%) (data not shown). In parallel, we studied the association between expression of chromogranin A and DLL3 (both categorized into positive or negative). A significant correlation was observed between the two markers (P = 0.024). DLL3 expression was observed in the majority (23 out of 26) of patients with positive chromogranin A (87.5%), while in patients with negative expression, DLL3 was found in 5 out of 10 cases (50.0%) (**Table 6**). As synaptophysin expression was available only for a subset of patients and was uniformly positive a meaningful correlation analysis between DLL3 and synaptophysin immunoreactivity was not possible.

**Table 4:** Univariate analysis of the association between presence of metastasis, site of disease and DLL3 expressions correlated with mOS.

Variables	Number of patients (%)	Number of deaths (%)	mOS in months (95% CI)	p-value (log-rank test)
Presence of metastasis				
No	34 (70.8)	34 (72.3)	4.1 (2.1-8.3)	0.019
Yes	13 (27.1)	12 (25.5)	9.8 (0.9-13.6)	
Unknown	1 (2.1)	1 (2.1)	-	
Site of disease				
GEP	14 (29.1)	14 (29.8)	4.0 (0.4-8.6)	0.076
Lung	24 (50.9)	23 (48.9)	7.8 (3.8-10.2)	
Head & neck	4 (8.3)	4 (8.5)	–	
Other*	4 (8.3)	4 (8.5)	–	
Unknown	2 (4.2)	2 (4.2)	–	
DLL3 expression				
Overall	48 (100.0)	47 (100.0)	6.6 (3.6-8.9)	0.067
Negative	12 (25.0)	12 (25.50)	7.3 (2.1-10.4)	
Low-positive	18 (37.5)	18 (38.3)	3.6 (0.9-8.3)	
High-positive	15 (31.3)	14 (29.8)	8.9 (0.5-13.4)	
Unknown	3 (6.3)	3 (6.4)	–	

In DLL3 expression: DLL3 negative cases were separated from positive ones before median calculation. Low-positive: lower than the median of positive cases; high-positive: higher than the median of positive cases. * comprises bladder and uterus.

**Table 5:** Univariable and multivariable Cox proportional hazards regression analyses of factors associated with mOS.

Variables	Univariable models		Multivariable model	
	HR (95%CI)	p-value	HR (95%CI)	p-value
Presence of metastasis				
No	1.00	0.022	1.00	0.008
Yes	2.27 (1.12-4.60)		3.32 (1.37-8.03)	
Site of disease				
GEP	1.00	0.082	1.00	0.903
Lung	0.53 (0.27-1.1)		0.95 (0.39-2.32)	
DLL3 expression				
Negative	1.00		1.00	
Low-positive	1.55 (0.74-3.24)	0.243	2.16 (0.84-5.56)	0.110
High-positive	0.63 (0.28-1.41)	0.262	0.42 (0.17-1.05)	0.065

Hazard ratios (HR), 95% confidence intervals (CIs), and p-values are reported.

**Table 6:** Relation between DLL3 expression and Ki-67 or chromogranin A.

Markers	DLL3 expression			
	Overall (%)	Negative (%)	Positive (%)	*p*-value (Fisher’s exact test)
Ki-67				
≤ 55%	8 (100.0)	2 (25.0)	6 (75.0)	0.456
> 55%	25 (100.0)*	4 (16.0)	21 (84.0)	
Chromogranin A				
Negative	10 (100.0)	5 (50.0)	5 (50.0)	0.024
Positive	26 (100.0)**	3 (11.5)	23 (88.5)	

* For 1 patient with Ki-67 > 55%, evaluation of DLL3 resulted not acceptable.

**For 2 patients positive for chromogranin A, evaluation of DLL3 resulted not acceptable.

## 4 Discussion

NECs are high-grade malignancies, biologically and clinically distinct from NETs, that pose significant clinical challenges due to the lack of sensitive prognostic markers and the limited availability of effective therapies beyond standard chemotherapy (30). In recent years, DLL3 has emerged as a promising biomarker and therapeutic target in lung NECs (31, 32). DLL3 blocks Notch signaling by retaining receptors in the Golgi apparatus (33), promoting tumor growth, migration, survival, immune microenvironment modulation, and activation of non-canonical pathways including AKT, Snail, and Wnt/β-catenin (34). Although DLL3 was first described in pulmonary NECs (23–27), we found DLL3 to be widely expressed also in GEP-NECs, distinguishing them from NETs (28). In the present study, we examined DLL3 expression in NECs from various anatomical sites to determine its prevalence and expression patterns. DLL3 was found to be highly expressed in NECs of all sites of origin, with an overall prevalence of 68.8%. These results confirm the findings of the analysis conducted by Lozada et al., where the authors reported a largely diffused DLL3 expression in poorly differentiated NECs, including extrapulmonary and GEP-localized tumors (35). The multicentric study of 1,294 pulmonary and extrapulmonary NENs by Schmitt et al. (36) further supports DLL3 specificity to NECs, showing a strong association with poorly differentiated histology. Taken together these findings highlight DLL3 as a potentially tumor marker and target in high-grade NECs, regardless of the primary site. In the present study, the highest prevalence was observed in GEP-NECs (85.7%), followed by lung NECs (58.3%). Previously reported rates for SCLCs range from 72.3% (37) to 94% (38), for LCNECs from 62.2% (36) to 74% (21), and for GEP-NECs from 54.8% (39) to 76.9% (28). Discrepancies likely reflect methodological differences and inter-laboratory variability, underscoring the need for prospective multicenter studies with harmonized criteria. DLL3 localization was predominantly cytoplasmic (60.6%), with combined membrane and cytoplasmic expression in 24.2% and exclusive membrane localization in 15.2% of cases, consistent with patterns described in lung NECs (40). An interesting observation was the dot-like pattern of DLL3 expression, particularly observed in NECs of gastrointestinal origin. This staining pattern may reflect localization within neurosecretory granules, as previously described in gastrointestinal mixed adenoneuroendocrine carcinomas (GI-MANECs), especially in chromogranin A–positive tumor cells (41). This suggests an underlying regulatory mechanism that warrants further investigation. DLL3 expression was found to be independent of Ki-67 proliferation index; however, it should be noted that Ki-67 assessment is not consistently available in lung NECs in our cohort, reflecting its non-routine assessment in clinical practice. DLL3 expression showed a correlation with chromogranin A, being positive in the majority of chromogranin A-positive cases and in 50% of chromogranin A-negative cases, supporting the assumption that chromogranin A does not detect all NEC cases, and underscoring the relevance of adding DLL3 evaluation in the diagnostic procedure of these tumors. The lack of variability in synaptophysin expression across our cohort precluded assessment of a potential association between synaptophysin and DLL3 expression. No statistically significant association was found between DLL3 expression and overall survival. This result may be influenced by the limited sample size of the cohort and confounding variables. Metastatic disease was the only independent predictor of worse mOS in univariable and multivariable models, underscoring its dominant prognostic role in this cohort. However, the result is consistent with other large-scale studies. In particular, Schmitt et al., did not establish an independent prognostic role for DLL3 in survival analysis in pulmonary and extrapulmonary NECs, although they found DLL3 expression to correlate with aggressive biology (36). Kuempers et al. found no significant difference in DLL3 expression between chemo-naïve (86.6%) and chemo-relapsed (80%) SCLC patients, though high DLL3 expression in relapsed samples trended toward better prognosis (42). A similar trend was observed in our cohort when dividing cases with high versus low DLL3 expression, based on the median level. This finding merits further exploration, as it may indicate a correlation between DLL3 expression levels and therapeutic sensitivity. Stratification according to the presence of metastasis at diagnosis in large case series are warranted. From a therapeutic standpoint, DLL3-targeted agents have become the focus of several clinical trials, especially in SCLC. Rovalpituzumab tesirine (Rova-T) showed promising results in early-phase trials (ORR 12–14%, mOS 5.6 months) (43) but failed in Phase III studies due to toxicity and limited efficacy (44). More recently, novel strategies have emerged, such as DLL3-targeted bispecific T-cell engagers (BiTEs) and CAR-T cell therapies, which aim to enhance specificity and reduce off-target effects. AMG 757 (tarlatamab), a half-life–extended (HLE) DLL3-targeted BiTE, recruits CD3+ T cells to DLL3-expressing tumor cells. Preclinical models showed strong antitumor activity with good tolerability (45). In the early clinical trial DeLLphi-300, tarlatamab demonstrated an ORR of 23.4 %, PFS of 3.7 months, and OS of 13.2 months in heavily pretreated SCLC patients (24) and significantly longer OS versus chemotherapy in the Phase 3 DeLLphi-304 trial (13.6 vs. 8.3 months) (26). Obrixtamig, another DLL3/CD3 bispecific, has shown promising activity particularly in high DLL3-expressing tumors (NCT04429087) (46). The high DLL3 expression in extrapulmonary NECs, as shown in our findings and other reports, provides a strong rationale to support further investigation of DLL3-directed therapeutic strategies into GEP-NECs, where prognosis remains poor and treatment options limited. This study has several limitations. First, the single-center design and relatively small sample size, inherent to the rarity and biological heterogeneity of NECs, limit the generalizability of our findings, which require validation in larger, independent cohorts. Consequently, the survival analyses should be considered exploratory and hypothesis-generating. In addition, the biological and clinical significance of the distinct localization patterns (membranous, cytoplasmic or dot-like) remains poorly understood. In particular, while DLL3-targeted therapies are expected to act on membrane-expressed antigen, the relationship between specific DLL3 staining patterns and response to anti-DLL3 therapies has yet to be established. Further studies are therefore needed to determine whether different patterns of DLL3 expression are associated with distinct therapeutic responses or clinical outcomes. Finally, although we observed an association between DLL3 and chromogranin A expression, the biological basis and potential clinical significance of this finding remain unclear and warrant further investigation. In summary, DLL3 expression appears to be widely diffused in high-grade NECs of different sites of origins and may serve not only as a diagnostic marker but also as a therapeutic target, particularly in GEP-NECs.

## 5 Conclusions

DLL3 might be useful to improve the diagnostic accuracy for NECs, especially in those cases where morphology does not adequately reflect the pathologic grade. Multicenter studies using harmonized immunohistochemical criteria or integrated molecular profiling are needed to define the expression landscape of DLL3 in heterogeneous disease groups. Finally, this study contributes to the growing evidence of using DLL3-targeting strategies in GEP-NECs, that will potentially lead to significant advancements in the management of patients affected by this aggressive disease.

## Data Availability

The original contributions presented in the study are included in the article/**Supplementary Material**. Further inquiries can be directed to the corresponding author.
